# Highly Stretchable, Self-Healing, Injectable and pH Responsive Hydrogel from Multiple Hydrogen Bonding and Boron-Carbohydrate Interactions

**DOI:** 10.3390/gels9090709

**Published:** 2023-09-01

**Authors:** Yi-Yang Peng, Qiuli Cheng, Meng Wu, Wenda Wang, Jianyang Zhao, Diana Diaz-Dussan, Michelle McKay, Hongbo Zeng, Sarute Ummartyotin, Ravin Narain

**Affiliations:** 1 Department of Chemical and Materials Engineering, University of Alberta, Edmonton, AB T6G 2G6, Canada; ypeng5@ualberta.ca (Y.-Y.P.); mwu4@ualberta.ca (M.W.); wenda@ualberta.ca (W.W.); ddiaz@ualberta.ca (D.D.-D.); mmckay@ualberta.ca (M.M.); hongbo.zeng@ualberta.ca (H.Z.); 2 School of Materials Science and Engineering, Henan University of Science and Technology, Luoyang 471023, China; chengql2012@163.com; 3 School of Biomedical Sciences and Engineering Guangzhou International Campus, South China University of Technology, Guangzhou 511442, China; jianyangzhao2017@gmail.com; 4 Department of Materials and Textile Technology, Faculty of Science and Technology, Thammasat University, Pathum Thani 12120, Thailand; 5 Center of Excellence on Petrochemical and Materials Technology, Chulalongkorn University, Bangkok 10330, Thailand

**Keywords:** self-healing, injectable, hydrogel, boron-carbohydrate interaction, pH responsiveness, diols responsiveness, glycopolymer, drug delivery

## Abstract

A simple and cost-effective method for the fabrication of a safe, dual-responsive, highly stretchable, self-healing and injectable hydrogel is reported based on a combination of dynamic boronate ester bonds and hydrogen bonding interactions. The mechanical properties of the hydrogel are tunable by adjusting the molar ratios between sugar moieties on the polymer and borax. It was remarkable to note that the 2:1 ratio of sugar and borate ion significantly improves the mechanical strength of the hydrogel. The injectability, self-healing and stretchability properties of the hydrogel were also examined. In addition, the impact of the variation of the pH and the addition of free sugar responsiveness of the hydrogel was studied. High MRC-5 cell viability was noticed by the 3D live/dead assay after 24 h cell culture within the hydrogel scaffold. Hence, the developed hydrogels have desirable features that warrant their applications for drug delivery, scaffolds for cell and tissue engineering.

## 1. Introduction

With the presence of hydrophilic groups, hydrogels are structurally constructed to create a three-dimensional (3D) crosslinked polymeric network containing high water content. Due to their large water trapping capability, similarity to tissue, tunable physiochemical properties and low cytotoxicity, hydrogels show great potential in various bioengineering applications [1,2,3]. Furthermore, their porous nature allows the transport various types of small molecules such as therapeutic agents, nutrients and oxygen, providing advantages in drug, gene and cell delivery [4,5,6,7,8,9]. However, conventional hydrogels that are crosslinked by covalent bonds are prone to damage even under normal operating conditions, which cannot satisfy the needs in medical technology [10].

More advanced or intelligent multifunctional hydrogels with self-healing and/or injectability were designed aiming to fulfill these requirements [10,11,12,13,14,15,16]. Injectable self-healing hydrogels can be constructed based on dynamic covalent bonds [17,18,19,20,21] or non-covalent interactions [20,21], imine bonds [22,23], disulfide bonds [24,25] and boronic esters [26,27,28,29,30,31] are the typical dynamic covalent bonds employed for preparing injectable self-healing hydrogels. Among these crosslinking strategies, the most interesting one is based on boronic esters. Our group designed multi-responsive and injectable hydrogels (pH, diols and oxidation) by using polyphenol [29] or glycopolymer [32] containing benzoxaborole residues. The difference in Rhodamine B (RhB) release profile in the presence of different triggering signals (diols and oxidizer) [29] provided the selective releasing property to the hydrogel. As a consequence, it can be used to investigate the accumulation of therapeutic agent at the target site. Furthermore, this platform can be guided to the design of the drug molecule and its delivery systems while providing comfort to the patient by minimizing pain given the injectability of the hydrogel [33,34,35]. Furthermore, it can be developed as a bio-ink for 3D printing formation for specific design in tissue engineering [36,37]. Besides their uses as therapeutic delivery tools, this smart-responsive degradable hydrogel can also be utilized in addressing the challenges of vascularization in tissue engineering [38,39,40]. Recently, Hsu et al. proposed the use of glucose-sensitive self-healing hydrogels as a sacrificial material for constructing the space for vascularization [41]. The glucose-sensitive hydrogel was extruded and placed in a conventional hydrogel. Subsequently, it was soaked in a culture medium to dissolve and remove the excess of glucose-sensitive hydrogel by dissolving it into the culture medium. The presence of sugar resulted in the creation of branched tubular channels in the conventional hydrogel. By using this technique with the encapsulation of vascular endothelial cells in glucose-sensitive hydrogel and neural stem cells in the conventional hydrogel, vascularization was achieved after 14 days. Therefore, the biocompatible and smart-responsive hydrogel holds great promise in overcoming the challenge of vascularization in tissue engineering. However, the sophisticated preparation steps [42] of benzoxaborole are considered a drawback for the hydrogel’s commercial exploitation. To address this, a commercially available agent, borax, was chosen to crosslink the glycopolymer. This chemical reagent was commonly employed as a food additive with the additional property of non-toxicity [43]. The crosslinking can be formed by the formation of covalent or hydrogen bonding with diol groups. After dissolution in an aqueous medium, borax dissociates into either trigonal boric acid (B(OH)_3_) or tetrahydroxy borate ion (B(OH)_4_^−^) [44,45,46,47,48]. The ratio depends strongly on the pH. It is important to note that it can form B(OH)_4_^−^ in the basic pH range of the solution and results in higher crosslinking density [49,50,51]. Our group has developed methods for preparation of 2-lactobionamidoethyl methacrylamide (LAEMA)-based glycopolymers. These glycopolymers are known for their ease of preparation and low cytotoxicity. These glycopolymers were found to be promising in drug/gene delivery, surface coatings, hydrogels and tissue engineering [28,32,52,53,54,55]. A hydrophilic polymer with sugar moieties was designed and prepared as the hydrogel precursor. Borax was used to crosslink the polymers by boron–carbohydrate interaction to form the hydrogel; in addition, because of an excess number of hydroxyl groups on the sugar moieties, hydrogen bonding further strengthen the mechanical properties of the formed hydrogel. As boron–carbohydrate interaction forms dynamic covalent bonds, the self-healing property and injectability are expected to be observed in the resulting hydrogels. The self-healing property, injectability and moldability of the hydrogels were studied. In addition, for examining the potency of the hydrogels in cell encapsulation, drug delivery and tissue engineering as a sacrificial material, the in vitro cell viability, responsiveness (pH and diol groups) and drug (RhB) release profile in an acidic and physiological environment were studied. From these results, we demonstrated that the developed hydrogels possess the needed characteristics that promise their usage in tissue engineering, drug delivery and cell encapsulation.

## 2. Results and Discussion

**Scheme 1:**
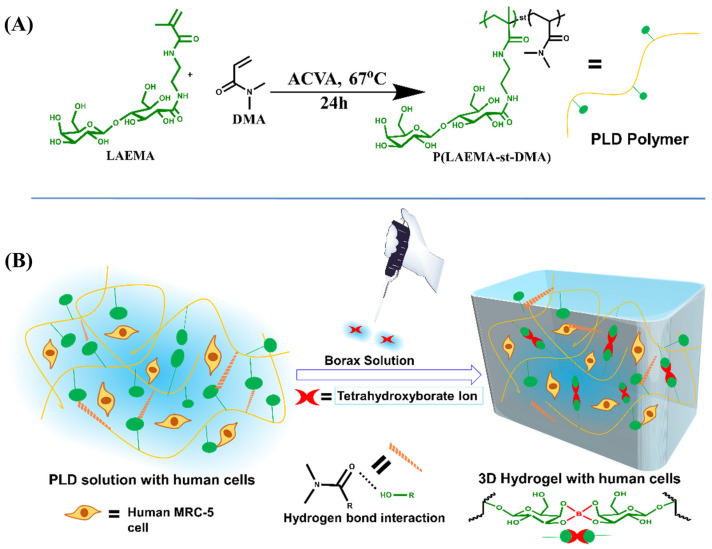
Graphical representations of the preparation of galactose-based polymer via free-radical polymerization (**A**) and the process of constructing a 3D matrix for cell encapsulation by adding borax as a gelation inducer (**B**).

As our group proposed to crosslink the polymer by using dynamic boron–carbohydrate interaction, and hydrogen bonding, a statistical glycopolymer, Poly(LAEMA-*st*—DMA) (PLD), was prepared via free-radical polymerization, and ACVA was used as an initiator (Scheme 1A). The success in making statistical and linear PLD was evidenced by GPC (Table S1) and ^1^H NMR (Figure S1). As determined by GPC, the molecular weight of PLD was ~59,000 kDa and the polydispersity was 7.7. The difference in reactivity between acrylate-based monomer (DMA) and methacrylate-based monomer (LAEMA) potentially contributes to the broad polydispersity of PLD [56]. The molar ratio between DMA and LAEMA was 9 to 1 according to the ^1^H NMR spectrum in D_2_O. To determine whether the PLD possesses potency to be used in smart-responsive drug delivery and as a sacrificial material for tissue engineering applications, the cell viability of PLD was evaluated by using a standard MTT assay with HeLa and MRC-5 cell lines (Figure S2). Good cell viability of PLD is important as it minimizes the damage to the surrounding tissue of the implantation and encapsulated cell after the degradation of the hydrogel. No significant cell death was observed with both cell lines for a polymer concentration at 1 mg/mL.

The general preparation process of hydrogel is shown in Scheme 1B. After the addition of 5.25 mL of 10 wt% borax (in PBS) into 0.3 mL 10 wt% PLD solution in PBS, a clear and transparent hydrogel formed in less than 5 s. As the glycopolymer has a large number of free hydroxyl groups, they can also form a large number of hydrogen bonds within the hydrogel network, thus this hydrogel is crosslinked based on two main types of interactions: hydrogen bonding interactions and boron ester dynamic covalent bonds.

The hydrogels were successfully formed at a pH 7.4, our added amount of tetrahydroxyborate ions was sufficient to form the hydrogel. The dynamic covalent bonds and the hydrogen bonding contribute to the self-healing properties of the hydrogel.

**Figure 1:**
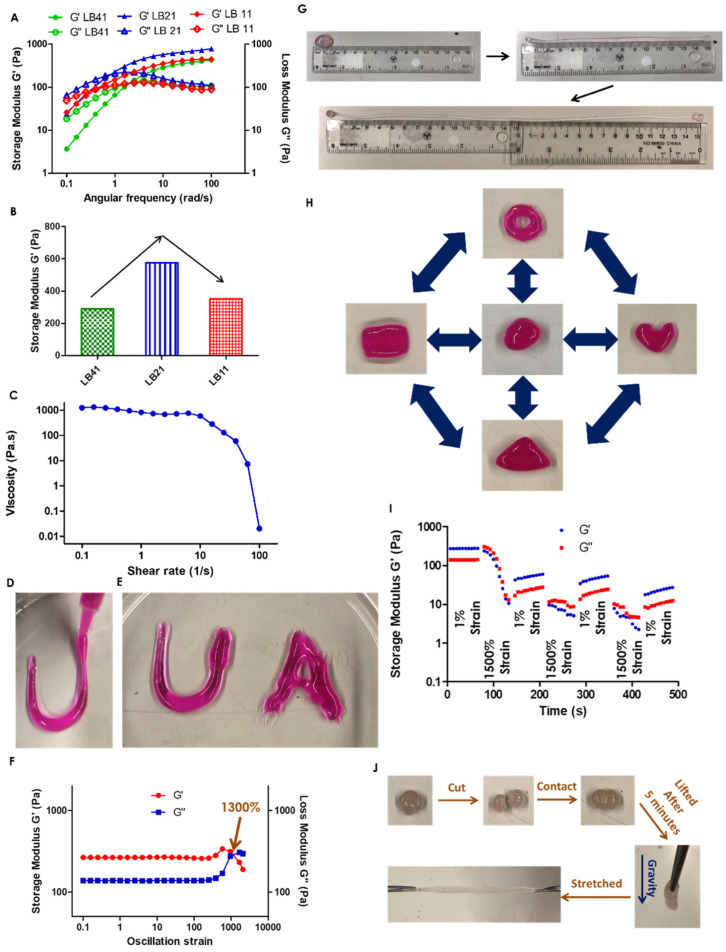
(**A**) Storage (G′) and loss (G″) modulus of LB 41, LB 21 and LB 11 as determined by dynamic oscillatory frequency sweep test (γ = 1%, ω = 1 Hz). (**B**) Storage modulus G′ of hydrogels (γ = 1%, ω = 1 Hz). (**C**) Shear-thinning property of hydrogel. (**D**) A visual image of the extrusion of hydrogel through a syringe. (**E**) Hydrogel-printed words “UA”). (**F**) Oscillation strain sweep of LB 21. (**G**) Visual demonstration of stretchability of LB 21. (**H**) Moldable property of LB 21. (**I**) Step-strain experiment of LB 21. (**J**) Illustration of self-healing property of LB 21.

According to the results of the dynamic oscillatory frequency sweep rheological experiments (Figure 1A), a frequency-dependent storage modulus (G′) and/or loss modulus (G″) was observed with all three prepared hydrogels. This dependency was expected as the hydrogels relied on dynamic covalent bond crosslinking [57,58]. According to the results, the highest G′ was obtained from LB 21 (Figure 1B), which provided more design parameters for the future design of this kind of hydrogel. LB 21 has a higher G′ than LB 41 and LB 11, which may be due to a higher crosslinking density. The ratio between sugar moieties and borax needs to be optimized. Without the optimization, an insufficient amount of tetrahydroxy borate ions or diols groups resulted in saturation, which leads to un-crosslinking of the polymer which lowers the value of G′. As boron–carbohydrate interaction is a dynamic bonding, self-healing and injectability are expected in the hydrogels. We decided to focus on conducting experiments with LB 21 to examine its self-healing and injectability properties by visual inspection and rheometer.

To study the injectability of LB 21, the shear-thinning property of LB 21 was examined. As the viscosity gradually decreased with the increase of shear rate (from 10 to 100/s), the shear-thinning property of LB 21 was confirmed (Figure 1C). To further confirm the injectability of LB 21, the extrusion of LB 21 through a syringe (Figure 1D) and printing of the abbreviation “UA” (Figure 1E) were conducted. By validating the injectability of LB 21, the success of LB 21 in the proposed applications (3D printing, drug delivery and tissue engineering) of the hydrogel become more achievable.

As mentioned before, traditional hydrogels are prone to damage. Self-healing properties can potentially address this challenge. Thus, the self-healing properties of LB 21 were examined. For determining the critical strain to break of LB 21, the oscillation strain sweep was applied to LB 21 from 0.1% to 2500%. LB 21 broke at 1300.0% as, at that strain, the G′ modulus started to be lower than G″ (Figure 1E). Because of its endurance at high strain, LB 21 is highly stretchable. To further confirm the stretchability of LB 21, LB 21 was extended from 2 cm to 30 cm (1400%), which was close to the value determined by the oscillation strain sweep (Figure 1G). Meanwhile, the easily moldable properties of LB 21 were also observed by molding LB 21 into the shapes of a ring, a sphere, a triangle, a heart and a rectangle. The stretchability and moldability allow the hydrogel to fit into various shapes of space and are useful for applications. A step-strain test with LB 21, that was conducted by rheometer, showed the self-healing properties of LB 21 as G′ was larger than G″ in the phase of small strain (1%) and vice versa at large strain (1500%) (Figure 1I). Loss of the hydrogel, because of the squeeze from the high strain test, contributed to the decrease of G′ in the first large (recovery) phase to the second cycle. Reformation of LB 21 was observed for up to three cycles as the value of the storage and loss modulus recovered partially in the phase of small strain. The recovery of value resulted from the reformation of dynamic covalent bonding and hydrogen bonding. The values were not fully recovered in the second and third cycles, which may be caused by less reformation of hydrogen bonding in that period. Visual observation was also conducted to examine the self-healing property of LB 21 (Figure 1J). A piece of LB 21 was cut into two pieces and subsequently made contact between two pieces of hydrogel for 5 min. After 5 min, the repaired hydrogel was lifted up and stretched.

**Figure 2:**
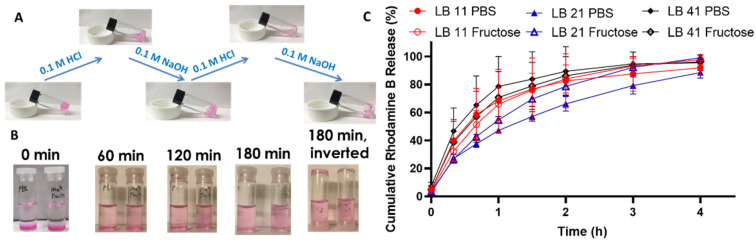
Visual illustrations of pH responsiveness of BL 21 by adding either 0.1 M HCl or NaOH to turn it to sol/gel phase (**A**), and stability of BL 21 in the presence of fructose in comparison with PBS alone (**B**). BL 21 was stained by using RhB. RhB release studies of (**C**) LB 11, LB 21, and LB 41 in PBS and fructose solution for 4 h.

**Figure 3:**
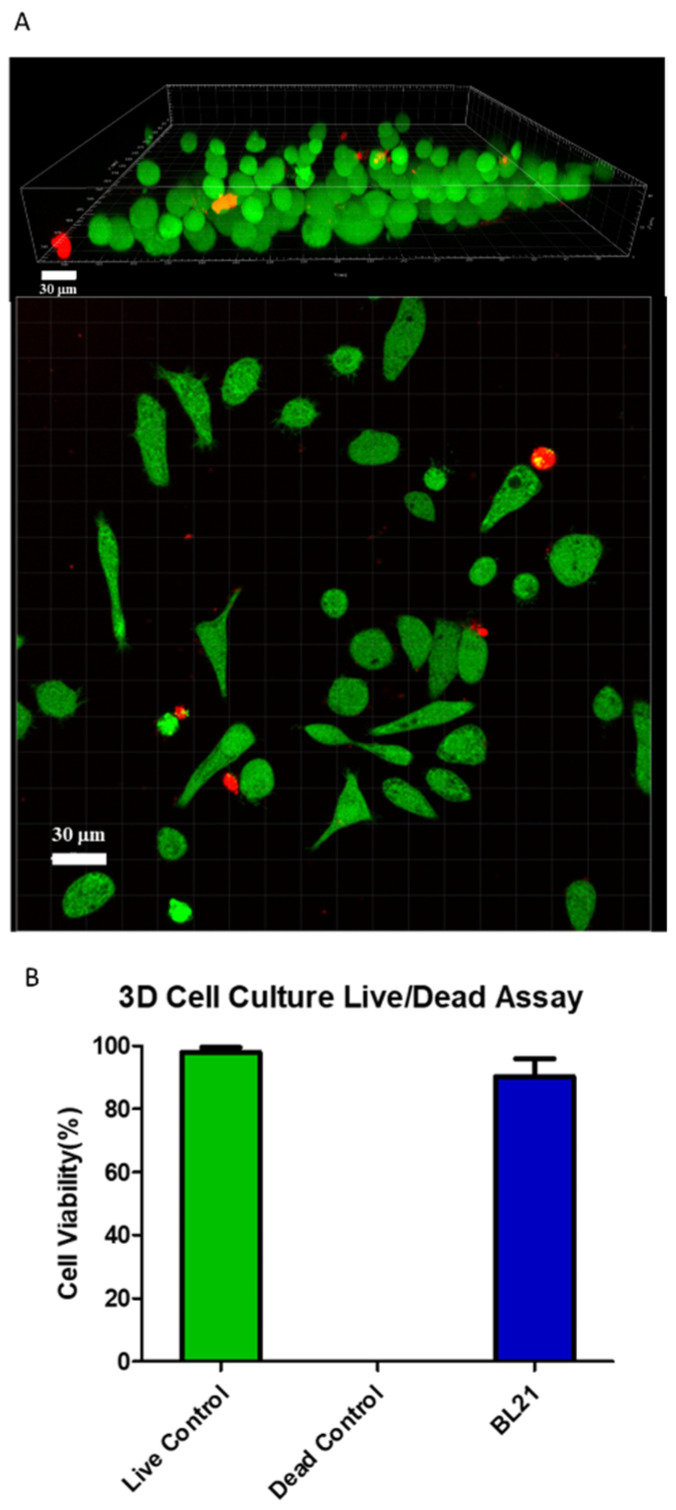
A 3D image of MRC-5 cells cultured in BL 21 for 24 h, as characterized by using 3D live/dead assay and demonstrated by using a fluorescence confocal microscope (**A**). Cells in green/red color indicate live/dead cells. Cell viability of the encapsulated MRC-5 cells (**B**).

As the boron–carbohydrate interaction is sensitive to diols and pH, the responsiveness of the hydrogel in the presence of free sugars and pH were investigated by visual illustration. Furthermore, RhB release profiles were further constructed in the presence of fructose and PBS to examine the potency of the hydrogels in drug delivery. By adding 0.1 M HCl to LB 21, LB 21 turned to sol phase, and LB 21 recovered to gel phase from sol phase after the addition of 0.1 M NaOH (Figure 2A). Two cycles were conducted. After three hours of incubation in 100 mM fructose in PBS solution, LB 21 was completely dissolved (Figure 2B). This observation proved that the diol responsiveness of LB 21 as the presence of fructose accelerated the degradation speed of LB 21. For determining the potency of this system in drug delivery, the RhB release profile in the PBS solution with or without the presence of fructose was made with LB 41, LB 21 and LB 11 (Figure 2C). Faster release of RhB was observed with all three hydrogels in the presence of fructose, which further confirmed the diol responsiveness of this system. The variation of release profile between hydrogels provides more opportunity to design the hydrogel to satisfy the needs of the target treatment by adjusting the molar ratio between LAEMA and borax. From the 3D live/dead assay with lung fibroblast cell lines (MRC-5) encapsulated in LB 21 for 24 h, the in vitro cell viability was 90.28 ± 5.58% (Figure 3A,B).

According to these results, injectable and sacrificial LB 21 facilitates the process of vascularization in a conventional hydrogel by forming space and cell transport. Furthermore, the high cell viability of LB 21 potentially allows it to minimize encapsulated vascular endothelial cell death, which addresses the challenge in tissue engineering. In comparison to our previous reports with benzoxaborole [28,29,32], we developed a more economical method for constructing material for cell encapsulation. Borax is an easily non-expensive obtainable reagent and the preparation of glycopolymer is well-developed in our laboratory. In addition, as hydrogen bonding was employed in LB 21, the stretchability of LB 21 is superior to our other previous report. In summary, hydrogels with pH and diol responsiveness, injectability, highly stretchable and self-healing properties were developed by using borax and glycopolymer. As indicated by the live/dead assay, the hydrogel showed great potency for use in 3D cell encapsulation as the 24 h cell viability of MRC-5 was 90.28 ± 5.58%. Considering the ease of fabrication and favorable properties, the developed hydrogels will be considered for more advanced in vivo studies for cell or tissue scaffolds and wound dressing applications.

## 3. Conclusions

Herein, a facile method to prepare highly stretchable, self-healable, biocompatible, injectable and dual (pH and sugar) responsive hydrogels is reported based on glycopolymer–boric acid interactions. Poly [LAEMA-*st*-*N*,*N*-dimethyl acrylamide (DMA)] was prepared and crosslinked with borax to form dynamic bonds in a phosphate buffer solution (PBS, pH 7.4). In addition, the large number of hydroxyl groups in the sugar moieties allows the formation of hydrogen bonding interactions. These interactions contribute to the self-healing and enhanced mechanical properties of the hydrogel. The mechanical property of the hydrogel can be tuned by the ratio between LAEMA and borax. The resulting hydrogels are injectable, self-healable, moldable and highly stretchable as examined by visual observation and rheometer. As boron–carbohydrate interaction was used to crosslink polymer, diol groups and fructose in this study, this accelerated the degradation of the hydrogels. These properties provide promising advantages for LB 21 to be applied to tissue engineering, especially for vascularization. In addition, the pH and diol responsiveness allow LB 21 to be used in on-demand drug delivery applications. Further, in vivo studies with LB 21 will be considered.

## 4. Materials and Methods

### 4.1. Materials

We prepared 2-lactobionamidoethyl methacrylamide (LAEMA) by following our previously reported procedure [59]. *N*,*N*-dimethylacrylamide (DMA), 4,4′-azobis (4-cyanovaleric acid) (ACVA), fructose and rhodamine B (RHB) were ordered from Sigma Aldrich (Oakville). Boric acid was ordered from Fisher Chemicals by Thermo Fisher Scientific. DMF was purchased from Caledon Laboratories Ltd. (Georgetown, ON, Canada). All of the chemical reagents were used as received without any further purification.

### 4.2. Poly(LAEMA-st-DMA) (PLD) Preparation

Poly(LAEMA-*st*-DMA) (PLD) was synthesized via free-radical polymerization. In brief, 743 mg DMA (7.50 mmol) and 501 mg LAEMA (1.07 mmol) were dissolved in 7.30 mL of deionized water. Subsequently, 0.5 mL DMF, with pre-dissolved 3 mg ACVA (10.7 mmol), was added to the solution. The mixture was reacted at 67 °C for 24 h after degassing with nitrogen gas for 30 min. After the reaction, the polymerization was terminated by air exposure. Then, it was purified via dialysis (6–8 kDa) for three days. The dry white solid of PLD was acquired by using a freeze dryer.

### 4.3. Hydrogel Fabrication and Characterization

A total of 10 wt% of PLD and 10 wt% borax was dissolved in PBS solution (pH 7.4). It was continuously stirred at 95 °C and was stored as stock solutions. A total of 0.1 mL of PLD solution was mixed with 5.25 mL of borax solution to prepare 10 wt% hydrogel. Hydrogels with different ratios of LAEMA:tetrahydroborate ion were prepared by adjusting the amount of borax solution that was added to the PLD solution.

### 4.4. Characterization

#### 4.4.1. The ^1^H Nuclear Magnetic Resonance (NMR)

A Varian 500 MHz spectrometer was used to acquire ^1^H NMR spectra of the PLD in D_2_O.

#### 4.4.2. Gel Permeation Chromatography (GPC)

Conventional gel permeation chromatography (GPC) was used to determine the average molecular weight (*M_n_*) and polydispersity (*M_w_*/*M_n_*) of the polymer. The composition of PLD was obtained by analyzing the results of *M_n_* and the molar ratio between LAEMA and DMA of PLD. 0.5 M sodium acetate/0.5 M acetic buffer was used as the eluent of the GPC system. The flow rate was adjusted to 1.0 mL/min. A TSK-gel G5000PWxl column (Tosoh Bioscience, Tokyo, Japan) was used. The calibration curve was created by using monodisperse Pullulan standards (6.8 to 404 kDa).

#### 4.4.3. Rheometer

To examine the mechanical properties of the hydrogel, hydrogels with different crosslinking percentages were prepared by adjusting the molar ratio between LAEMA and borax. LB 41, LB 21 and LB 11 indicated the molar ratios between LAEMA and borax were at 4:1, 2:1 and 1:1, respectively (Table 1). The rheological and self-healing properties of hydrogel were characterized by at 25 °C using an AR-G2 rheometer (TA instruments) with a 20 mm 2.008° cone plate geometry. Frequency sweeps (from 0.1 to 100 rad/s with constant strain at 1%) were used to evaluate the mechanical properties of LB 41, LB 21 and LB 11. Critical strain for breaking of the LB 21 was determined by using oscillatory strain amplitude sweeps (0.1% to 2500.0%). The obtained critical strain was used further to study the self-healing properties of LB 21 by step-strain tests. In the step-strain test, the mechanical properties were recorded while applying a large strain (1500%) to break LB 21 and subsequently applying a small strain (1%) to allow the repair of LB 21. The cycle was repeated three times. Visual observation was also utilized to examine the self-healing properties of BL21. One LB 21 was cut into two pieces and the two pieces were allowed to contact each other for a minute, followed by lifting of the whole pieces up and stretching. For examining the moldable property, the hydrogel was molded into various shapes including a donut, square, sphere, heart and triangle. For studying the injectability of the hydrogel, LB 21, stained with Rhodamine B, was loaded into a syringe to print the words “UA”. For corroborating the shear-thinning property of BL21, the viscosity of the hydrogel as a function of shear rate (0.1 to 100/s) was recorded.

### 4.5. Responsiveness of Hydrogel

The responsiveness of BL21 was studied by the addition of free sugar, fructose. The RhB-stained BL21 was incubated with either 2 mL of PBS pristine solution or 50 mM fructose in PBS. Images were recorded at different time intervals. A Jasco V-630 UV-visible spectrometer was used to obtain the concentration of RhB (555 nm) in the incubating solution to construct the RhB release profile of the hydrogel. The pH responsiveness of BL21 was confirmed by adding 0.1 M HCl to turn the hydrogel into a solution and subsequently reforming the gel by adding 0.1 M NaOH. The cycle was repeated three times in order to ensure accuracy.

### 4.6. Cell Culture

HeLa and MRC-5 cells were grown at 37 °C under 5% CO_2_ in a humidified environment. DMEM medium with 10% fetal bovine serum (FBS) and 1% antibiotic (50 units of penicillin, 50 μg streptomycin) was used to supply nutrients to both cell lines. Subculture of cells was conducted when the confluency of cell reached 80%. A amount of 0.25% trypsin-EDTA was used to detach the cells and the frequency of this operation was two times per week.

### 4.7. Cytotoxicity of PLD

The cytotoxicity of PLD with HeLa and MRC-5 cell lines was evaluated by standard MTT assay. A total of 10,000 cells of either HeLa or MRC-5 cells were seeded into each well of a 96-well plate. The experiment was conducted overnight incubation in order to make cells adhere to the surface of the well. On the next day, new low glucose media with different concentrations of PLD (0.01 to 1.00 mg/mL) were used to replace the old medium, and the incubation period was 24 h. A total of 15 mL of 5 mg/mL MTT solution was added to each well after the incubation. A total of 4 h of incubation was conducted after the addition of MTT solution. The mixture of DMSO and isopropanol (1:1 of volume ratio) was used to dissolve the resulting purple crystal. The absorbance of each well was read by using a TECAN microplate at 570 nm.

### 4.8. The 3D Live/Dead Assay

MRC-5 cells were used to evaluate the 3D cell viability of BL21. UV light exposure was used to sterilize PLD and borax for 30 min. A total of 0.6 mL low glucose DMEM medium with pre-suspended MRC-5 cells (2.5 × 10^6^ cells/mL) was used to suspend 60 mg of PLD in a round glass-bottomed Petri dish. It was continuously stirred until homogeneity. Then, 10.5 mL of 10 wt% borax in PBS solution was poured into the sample to form the BL21. After that, it was incubated for 20 min, 2 mL of low glucose in DMEM medium was added to the dish. The cells in the hydrogel were stained by using the live/dead assay kit after 24 h of incubation at 37 °C. The live (green) and dead (red) cells were visualized by using a CLSM 710 Meta confocal laser scanning microscope (Carl Zeiss, Jena, Germany). Quantitative analysis and image processing were completed by using Imaris Image analysis software [60].

## Figures and Tables

**Table 1:** Composition of LB 41, LB 21 and LB 11.

Hydrogel	10 wt% PLD (mL)	10 wt% Borax (μL)
LB 41	0.3	2.63
LB 21	0.3	5.25
LB 11	0.3	10.5

## Data Availability

The data supporting the findings of this manuscript are available from the corresponding authors upon reasonable request.

## Supplementary Materials

The following supporting information can be downloaded at: https://www.mdpi.com/article/10.3390/gels9090709/s1, Figure S1: ^1^H NMR of PLD in D_2_O; Figure S2: Cell viability of the polymer, PLD, with HeLa and MRC-5 cell lines; Table S1: Composition, average molecular weight and polydispersity of PLD.
