# Supplementary material for: Highly Stretchable, Self-Healing, Injectable and pH Responsive Hydrogel from Multiple Hydrogen Bonding and Boron-Carbohydrate Interactions
Source: Gels. 2023 Sep 1;9(9):709. doi: 10.3390/gels9090709 (PMC10530767; doi:10.3390/gels9090709)
Supplement: Supplementary file 1 [file gels-09-00709-s001.zip › gels-2494559-supplementary.pdf]

Supplementary Information

Highly Stretchable, Self-healing, Injectable and pH responsive Hydrogel from Multiple Hydrogen Bonding and Boron-Carbohydrate Interactions

Yi-Yang Peng[†,‡], Qiuli Cheng [‡ ,‡], Meng Wu[†], Wenda Wang[†], Jianyang Zhao,[Π] Diana Diaz-Dussan[†], Michelle McKay[†], Hongbo Zeng[†], Sarute Unmartyotin[$,*] and Ravin Narain[†,*]

†Department of Chemical and Materials Engineering, University of Alberta, Edmonton, Alberta T6G 2G6, Canada.

‡School of Materials Science and Engineering, Henan University of Science and Technology, Luoyang 471023, P. R. China, .

[$]Department of Materials and Textile Technology, Faculty of Science and Technology, Thammasat University, Pathum Thani, 12120, Thailand.

[Π]School of Biomedical Sciences and Engineering Guangzhou International Campus, South China University of Technology, Guangzhou 511442, China

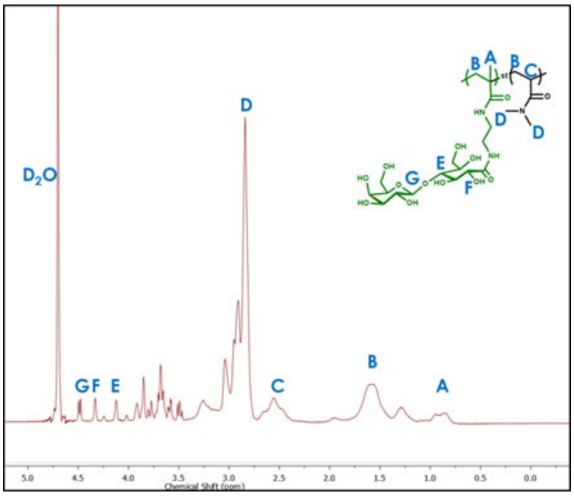

Figure S1. ^{1}H NMR of PLD in D_2O.

Table S1. Composition, average molecular weight and polydispersity of PLD.

Polymer	Composition (mol%)		Molecular Weight	
PLD	LAEMA	DMA	Mn (kDa)	PDI (Mw/Mn)
	10.0	90.0	59.0	7.68

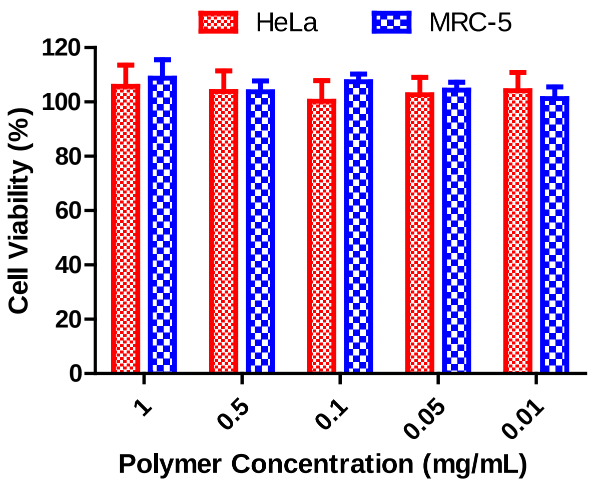

Figure S2. Cell viability of the polymer, PLD, with HeLa and MRC-5 cell lines.
